# Identification of differentially methylated genes in first-trimester placentas with trisomy 16

**DOI:** 10.1038/s41598-021-04107-9

**Published:** 2022-01-21

**Authors:** Ekaterina N. Tolmacheva, Stanislav A. Vasilyev, Tatiana V. Nikitina, Ekaterina S. Lytkina, Elena A. Sazhenova, Daria I. Zhigalina, Oksana Yu. Vasilyeva, Anton V. Markov, Victoria V. Demeneva, Liubov A. Tashireva, Anna A. Kashevarova, Igor N. Lebedev

**Affiliations:** 1 Research Institute of Medical Genetics, Tomsk National Research Medical Center, Tomsk, Russia; 2 National Research Tomsk State University, Tomsk, Russia; 3 Cancer Research Institute, Tomsk National Research Medical Center, Tomsk, Russia

**Keywords:** Developmental biology, Genetics

## Abstract

The presence of an extra chromosome in the embryo karyotype often dramatically affects the fate of pregnancy. Trisomy 16 is the most common aneuploidy in first-trimester miscarriages. The present study identified changes in DNA methylation in chorionic villi of miscarriages with trisomy 16. Ninety-seven differentially methylated sites in 91 genes were identified (false discovery rate (FDR) < 0.05 and Δβ > 0.15) using DNA methylation arrays. Most of the differentially methylated genes encoded secreted proteins, signaling peptides, and receptors with disulfide bonds. Subsequent analysis using targeted bisulfite massive parallel sequencing showed hypermethylation of the promoters of specific genes in miscarriages with trisomy 16 but not miscarriages with other aneuploidies. Some of the genes were responsible for the development of the placenta and embryo (*GATA3-AS1, TRPV6, SCL13A4,* and *CALCB*) and the formation of the mitotic spindle (*ANKRD53*). Hypermethylation of *GATA3-AS1* was associated with reduced expression of GATA3 protein in chorionic villi of miscarriages with trisomy 16. Aberrant hypermethylation of genes may lead to a decrease in expression, impaired trophoblast differentiation and invasion, mitotic disorders, chromosomal mosaicism and karyotype self-correction via trisomy rescue mechanisms.

## Introduction

The first trimester of pregnancy is the most critical period for embryo survival. At 8–10 weeks of pregnancy, cytotrophoblast cells invade the decidualized endometrium, results in remodeling of the spiral arteries of the uterus. Disruption of normal trophoblast differentiation and development and gestational remodeling of the uterine spiral arteries leads to a wide range of obstetric complications, from delayed fetal growth to fetal death and/or preeclampsia.

Mosaic trisomy may cause serious defects in physical and cognitive development^1^, which indicates that chromosomal aneuploidy has a harmful pleiotropic effect. The increased genes on the additional chromosome itself causes a genome-wide imbalance in gene activity. Aneuploidies of different chromosomes change the epigenetic profile of various tissues, including the placenta^2^.

Trisomy of chromosome 16 is the most common aneuploidy in miscarriages of the first trimester of pregnancy, and it accounts for approximately 6% of all miscarriages^3^. Most miscarriages with trisomy 16 are empty fetal sacs or disorganized embryos with minimal embryonic development^4^. Miscarriages with trisomy 16 also have abnormal chorionic villous morphology with a cystic, clubbed, or hypoplastic appearance. Mosaic forms of this aneuploidy occur later in pregnancy and postnatally in rare cases^5^. Mosaic forms of trisomy 16 are associated with a high probability of fetal death, preterm birth, intrauterine growth retardation, fetal abnormalities, and preeclampsia^6^. These malformations likely occur due to the aberrant expression of imprinted genes located on chromosome 16^7,8^.

Aneuploidies are also often characterized by a disturbance of the genome methylation profile, which leads to developmental disorders and embryonic death^2^. Epigenetic modifications in the placentas of fetuses with mosaic trisomy 16 and their relationship with early-onset preeclampsia were studied in detail previously^9^. However, data on DNA methylation in trisomy 16 in the first trimester of pregnancy, during which there is active placental differentiation and invasion of endovascular cytotrophoblasts into the spiral arteries, are scarce.

Trisomy 16 in first-trimester miscarriages primarily originates from segregation errors in maternal meiosis I^10^. Therefore, the formation of the body's epigenetic program after conception occurs against a background of trisomy 16 in the zygote and later in most cells, even in chromosomal mosaicism. Aneuploidy in this case is a causative factor for the induction of epigenetic changes in the genome. The present study identified differentially methylated genes in chorionic villi of first-trimester miscarriages with trisomy 16.

## Results

The study was performed in two stages. In the first stage, we performed a large-scale analysis of the DNA methylation profile in chorionic villi in 14 miscarriages with trisomy 16 and 6 induced abortions with a normal karyotype using the Infinium HumanMethylation27 BeadChip (Illumina). This stage identified numerous genes with differentially methylated CpG sites in miscarriages with trisomy 16. Using targeted bisulfite massive parallel sequencing, we analyzed the level of DNA methylation of the promoter regions of 5 differentially methylated genes on the expanded samples of miscarriages with trisomy 16 (n = 29). Only miscarriages with a normal karyotype and trisomy on other chromosomes were included in the study at this point because we were interested in the specificity of identified differential methylation of these genes for trisomy 16.

Samples of extraembryonic tissues of miscarriages and induced abortions were obtained from the biocollection of the Research Institute of Medical Genetics, Tomsk NRMC. These tissues were collected for 15 years and stored at a temperature of −80 °C. The criteria for inclusion in the study were the karyotype of miscarriage and gestational age from 6 to 12 weeks. The age of the parents was not specifically controlled.

### Genome-wide DNA methylation in chorionic villous trophoblasts with trisomy 16

**Figure 1:**
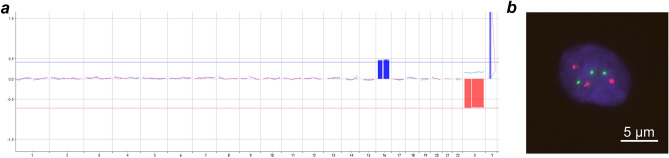
Results of cytogenetic analysis of chorionic villous trophoblasts of miscarriages with trisomy 16. (**a**) Example of the aCGH profile in chorionic villous trophoblasts with trisomy 16. **(b)** Example of chorionic villous trophoblasts with trisomy 16 based on the results of FISH analysis with two-color subtelomeric DNA probes specific to 16p (red) and 16q (green). Interphase nuclei were stained with DAPI (blue).

Trisomy 16 was discovered in chorionic villous trophoblasts of miscarriages using conventional karyotyping and array comparative genomic hybridization (aCGH) and validated further using interphase fluorescent in situ hybridization (FISH) (Fig. 1). The DNA methylation indices were analyzed in 27,578 CpG sites of 14,523 genes in chorionic villi of 14 miscarriages with trisomy 16 and 6 induced abortions with normal karyotypes. With the cutoff false discovery rate (FDR) < 0.05, 172 hypomethylated and 777 hypermethylated CpG sites were identified in miscarriages with trisomy 16. CpG sites with a Δβ > 0.15 were considered biologically significant differentially methylated sites (DMSs). With this cutoff, 2 hypomethylated sites in 1 gene and 94 hypermethylated CpG sites in 90 genes were identified. Hypermethylation of multiple CpG sites was detected in 4 genes (Supplementary Table S1). Twenty-three of these sites were located inside the CpG islands and 71 were not, but most of the differentially methylated sites were located in the regulatory regions of the genes. No chromosome-specific DMS enrichment was found, and only 4 differentially methylated genes (DMGs) were located on chromosome 16, which corresponded to the average level on other chromosomes (Supplementary Fig. S1).

We performed enrichment analysis of the 90 DMGs and found significant enrichment of a cluster of 47 genes encoding secreted proteins (p = 2.80E−08), signaling peptides (p = 6.50E−08), and receptors with disulfide bonds (p = 2.20E−06) (DAVID, UniProt, enrichment score: 8.76). Twenty-four of these genes were included in all 3 functional groups (Supplementary Fig. S1).

**Figure 2:**
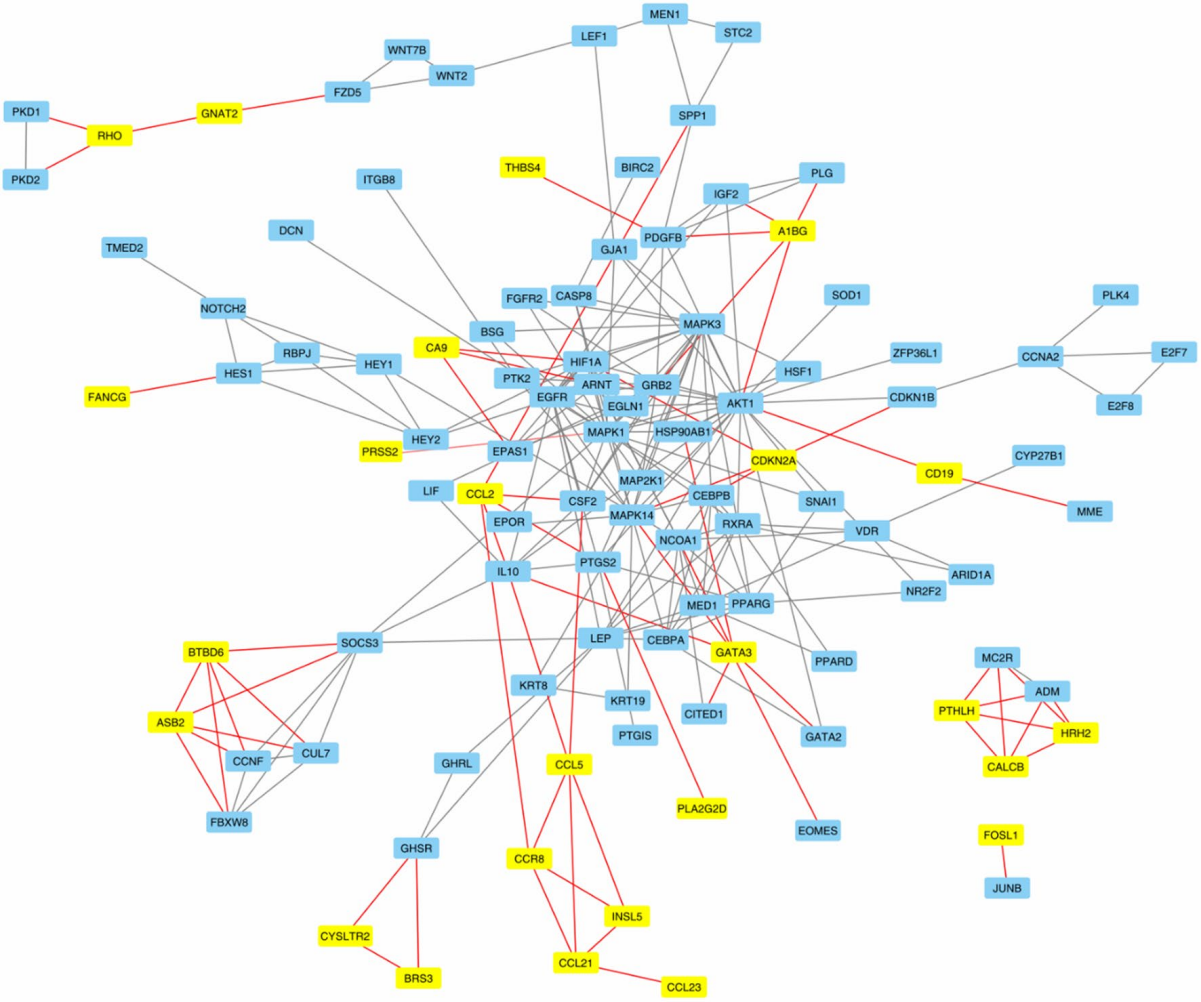
Functional relationships between the products of genes hypermethylated in chorionic trophoblasts of embryos with trisomy 16 (highlighted in yellow) and the products of genes involved in the development of the placenta (placental development, GO: 0001890) (highlighted in blue) (STRING database, score > 0.90). The connections of the products of the hypermethylated genes with the proteins involved in the development of the placenta are highlighted in red.

Among the DMGs in miscarriages with trisomy 16, two genes (*FOSL1* and *PCDH12*) were involved in placental development (GO:0001890). When the DMGs in miscarriages with trisomy 16 and genes involved in placental development (GO:0001890) were analyzed using STRING, 25 DMGs had functional connections with genes involved in placental development (score > 0.90, Fig. 2).

For a more rigorous identification of biologically significant DMGs, we used a criterion of Δβ > 0.2 and revealed hypermethylation in the promoters of 17 genes and hypomethylation of 1 gene (Table 1).

**Table 1:** Differentially methylated genes (FDR cutoff of p < 0.05 and Δβ > 0.2) in chorionic villi of miscarriages with trisomy 16.

Symbol	RefSeq	Product	CpG-island	Δβ	Chr
*ZNF804B*	NM_181646.2	Zinc-finger protein 804B	True	0.279	7
*ANKRD53*	NM_024933.2	Ankyrin repeat domain-containing protein 53	True	−0.275	2
*TRPV6*	NM_018646.2	Transient receptor potential cation channel; subfamily V; member 6	False	−0.268	7
*SEC31L2*	NM_015490.3	*S. cerevisiae* SEC31-like 2 isoform a	True	−0.258	10
*CCL2*	NM_002982.3	Small inducible cytokine A2 precursor	False	−0.233	17
*GATA3-AS1*	NM_207423.1	GATA3 antisense RNA 1	True	−0.226	10
*SLC13A4*	NM_012450.2	Solute carrier family 13 (sodium/sulfate symporters); member 4	False	−0.223	7
*SLC17A4*	NM_005495.1	Solute carrier family 17 (sodium phosphate); member 4	False	−0.221	6
*CYSLTR2*	NM_020377.2	Cysteinyl leukotriene receptor 2	False	−0.219	13
*ZNF683*	NM_173574.1	Zinc-finger protein 683	False	−0.216	1
*CALCB*	NM_000728.3	Calcitonin-related polypeptide; beta	True	−0.215	11
*PDZD3*	NM_024791.2	Sodium-phosphate cotransporter IIa C-terminal-associated protein 2	False	−0.214	11
*CCR8*	NM_005201.2	Chemokine (C–C motif) receptor 8	False	−0.210	3
*FANCG*	NM_004629.1	Fanconi anemia; complementation group G	True	−0.209	9
*KRTAP10-8*	NM_198695.1	Keratin-associated protein 10–8	False	−0.204	21
*BRS3*	NM_001727.1	Bombesin-like receptor 3	False	−0.203	X
*CCL21*	NM_002989.2	Small inducible cytokine A21 precursor	False	−0.202	9
*SLC3A1*	NM_000341.2	Solute carrier family 3; member 1	False	−0.201	2

### Detailed analyses of the DNA methylation of the identified DMGs

We analyzed the methylation indices of the promoter regions of 5 DMGs (*ANKRD53, TRPV6, GATA3-AS1, SCL13A4,* and *CALCB*) in chorionic villi trophoblasts of miscarriages with trisomy 16 and induced abortions with a normal karyotype using targeted bisulfite massive parallel sequencing (Table 2). Differentially methylated sites were located in the CpG islands for 3 genes: *ANKRD53, GATA3-AS1,* and *CALCB*.

**Table 2:** Localization and methylation of CpG sites of 5 differentially methylated genes analyzed by targeted bisulfite massive parallel sequencing in chorionic villi of miscarriages with trisomy 16.

Region	Strand	Genomic coordinates (hg19)	CpG	DMS	DMS/all CpG sites, %
*ANKRD53_1*	+	chr2: 71206056–71206605	40	6	15
		ANKRD53 in total	40	6	15
*CALCB_1*	+	chr11: 15094027–15094302	15	3	20
*CALCB_3*	+	chr11: 15094516–15094823	8	4	50
*CALCB_4*	+	chr11: 15094798–15095066	12	4	33
		CALCB in total	35	11	31
*GATA3_1*	+	chr10: 8091317–8091687	30	0	0
*GATA3_2*	+	chr10: 8092469–8093016	57	0	0
*GATA3_4*	+	chr10: 8093670–8094118	37	21	57
*GATA3_5*	+	chr10: 8094186–8094771	32	23	72
*GATA3_8*	+	chr10: 8095096–8095448	15	3	20
		GATA3-AS1 in total	84	47	56
*SLC13A4_1*	−	chr7: 135412984–135413410	11	0	0
*SLC13A4_2*	−	chr7: 135412563–135413021	7	6	86
		SCL13A4 in total	18	6	33
*TRPV6_1*	−	chr7: 142583430–142583865	5	0	0
*TRPV6_2*	−	chr7: 142582950–142583429	11	6	54
*TRPV6_3*	−	chr7: 142582830–142583240	6	5	83
		TRPV6 in total	22	11	50

Hypermethylation of various fractions of the analyzed CpG sites in the promoters of all 5 genes in miscarriages with trisomy 16 was found (p < 0.05) (Table 2). No hypomethylated CpG sites were observed. Therefore, the results obtained from targeted bisulfite massive parallel sequencing confirmed the results of the genome-wide DNA methylation analysis, which was performed using the Infinium HumanMethylation27 BeadChip.

**Figure 3:**
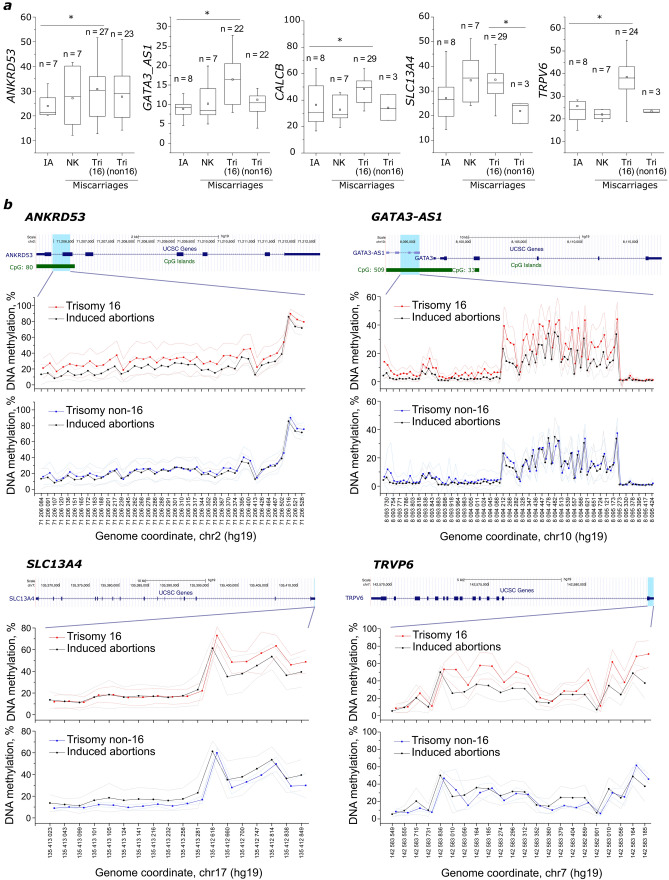
The CpG site methylation indices in 5 DMGs in chorionic villi in the group of miscarriages with trisomy 16 compared to miscarriages with trisomy of other chromosomes, miscarriages with a normal karyotype, and induced abortions. (**a**) The average methylation indices for all analyzed CpG sites in the *ANKRD53, GATA3-AS1, CALCB, SCL13A4,* and *TRPV6* genes in the groups of miscarriages with trisomy 16 (Tri (16)), miscarriages with trisomy of other chromosomes (Tri (non16)), miscarriages with a normal karyotype (NK), and induced abortions (IAs). The line in the center of the box marks the median. The boxes mark the 25th and 75th percentiles. The whiskers extend to the minimum and maximum values. The Mann–Whitney U test was used to compare groups. *—p < 0.05. (**b**) A detailed profile of the methylation of CpG sites in the analyzed regions of the *ANKRD53, GATA3-AS1, SCL13A4*, and *TRPV6* genes (indicated by a blue shaded area) in the groups of miscarriages with trisomy 16 and miscarriages with trisomy of other chromosomes compared to the induced abortion group. CpG islands are indicated by green bars. The dotted lines depict the standard deviation of the methylation profile. The figure was created using UCSC Genome Browser^11^.

The increased methylation indices of the studied genes may also be associated with aneuploidy on other chromosomes. To exclude this effect, we compared the methylation indices of the *ANKRD53* and *GATA3-AS1* genes in 26 miscarriages with aneuploidy other than trisomy 16 (trisomy 2, 7, 9, 11, 13, 14, 15, 18, 20, 21, or 22 or monosomy of chromosome 13 or the X chromosome) and 8 induced abortions with a normal karyotype. A similar comparison was made for the *TRPV6*, *SCL13A4*, and *CALCB* genes with miscarriages with trisomy 2 (n = 1) and trisomy 22 (n = 2). Only 1 hypermethylated site was identified for *GATA3-AS1*, and no DMS was found for *ANKRD53* (Fig. 3).

### Dependence of the gene methylation indices on gestational age and chromosome mosaicism

**Figure 4:**
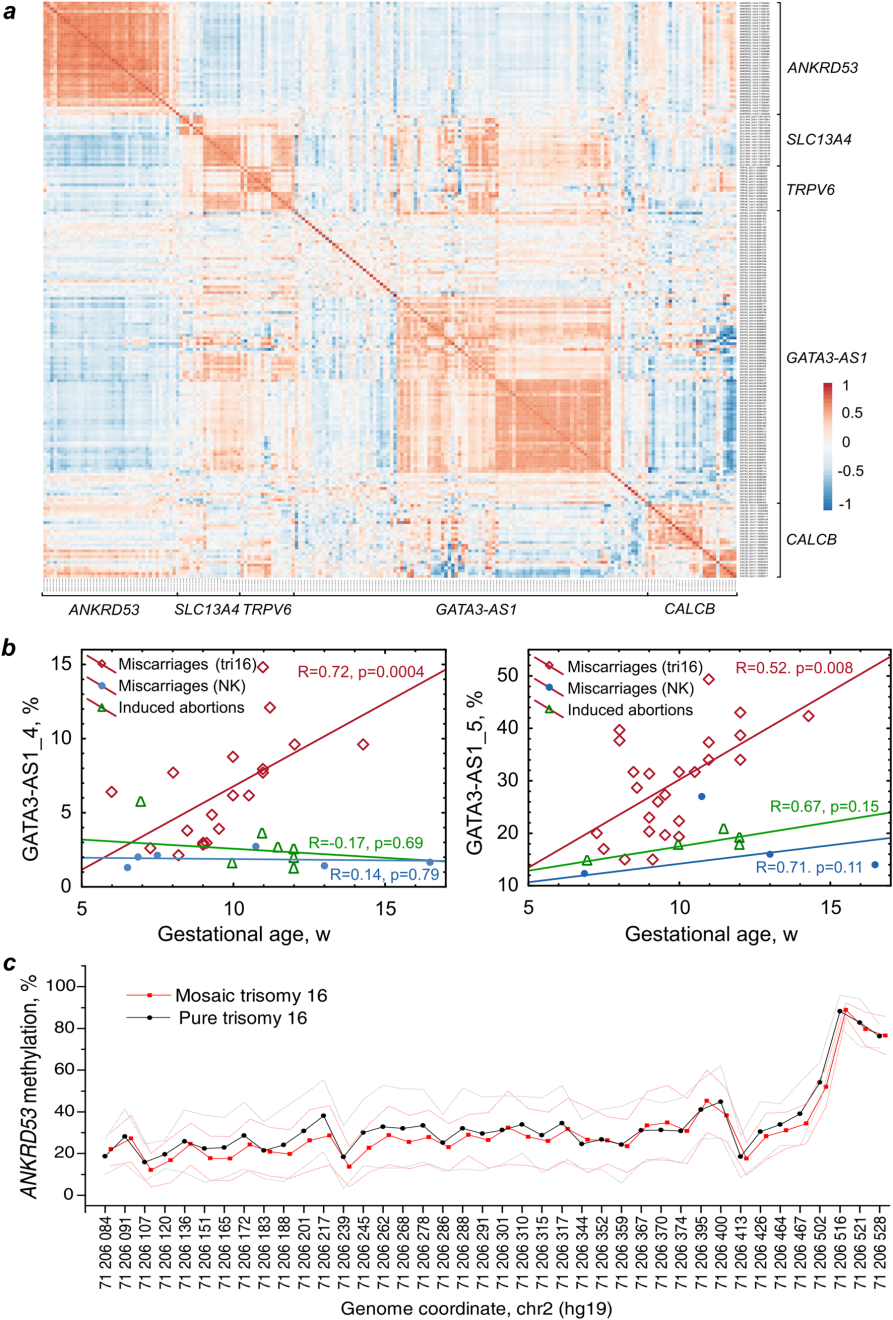
Correlation of the methylation indices of differentially methylated gene promoters with each other and their dependence on gestational age and chromosome mosaicism. (**a**) Correlation of the methylation indices of the *ANKRD53, TRPV6, GATA3-AS1, SCL13A4,* and *CALCB* genes with each other in chorionic villi of spontaneous abortions with trisomy 16. The heatmap was generated using the Spearman test and the ClustVis tool. (**b**) Correlation of the methylation indices of the GATA3-AS1_4-5 regions with the gestational age of miscarriages with trisomy 16, miscarriages with a normal karyotype, and induced abortions. (**c**) Comparison of the *ANKRD53* gene methylation index in chorionic villi between groups of miscarriages with mosaic and pure trisomy 16.

We analyzed the correlations between the gene methylation indices of 5 DMGs: *ANKRD53, TRPV6, GATA3-AS1, SCL13A4,* and *CALCB*. The methylation indices of the *GATA3*-*AS1*, *SCL13A4*, and *TRPV6* genes significantly correlated with each other (Fig. 4).

The methylation profile of the normal placenta changes with increasing gestational age^12^. Therefore, we analyzed the dependence of the CpG site methylation indices of all 5 DMGs in miscarriages with trisomy 16 on gestational age. A positive correlation was observed between gestational age and the level of GATA3-AS1_4-5 region methylation (Fig. 4).

The level of mosaicism of trisomy 16 was analyzed in some of the miscarriages (n = 30). Eighteen embryos had a pure form of trisomy 16, and the other embryos (n = 12) had mosaicism of trisomy 16, with a percentage of aneuploid clones ranging from 10 to 90%. The most hypermethylated gene in chorionic villi of miscarriages with trisomy 16 (Δβ = 0.27, Table 1), *ANKRD53*, encodes a protein that is involved in the mechanism of chromosome segregation in mitosis and may cause mitotic nondisjunction, which leads to chromosome mosaicism. However, the methylation indices of the *ANKRD53* gene promoter, and the promoters of the other four genes, did not differ between the groups with mosaic and pure forms of trisomy 16 (Fig. 4). This finding may be explained by the fact that trisomy 16 miscarriages have a predominantly meiosis I origin. Therefore, aneuploid–diploid mosaicism in embryos with trisomy 16 results from a postzygotic trisomy rescue mechanism. These results suggest that human embryos with trisomy 16 activate epigenetic programs to induce their own karyotype self-correction.

**Figure 5:**
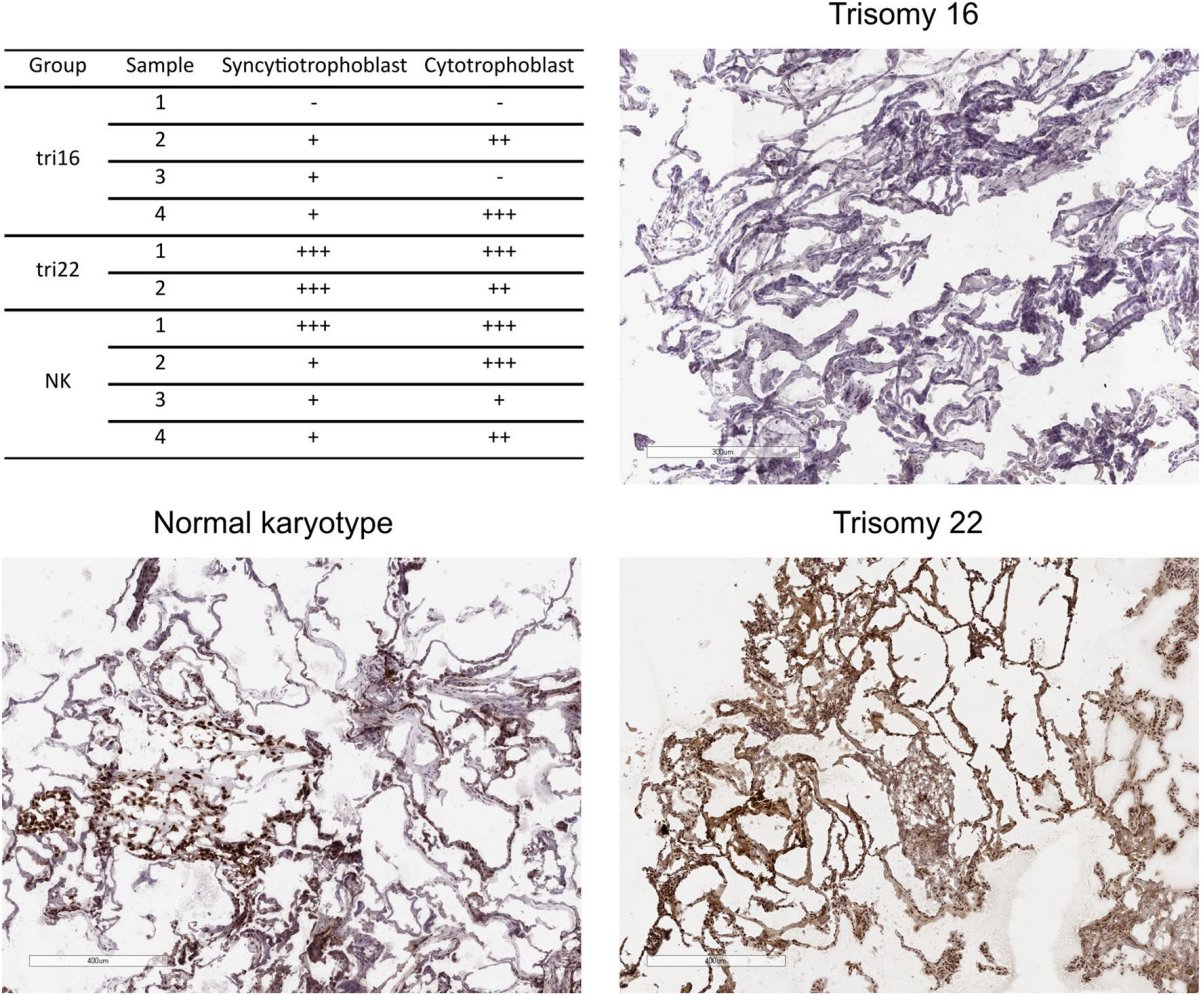
Examples of GATA3 protein staining in frozen sections of syncytiotrophoblast and cytotrophoblast cells of miscarriages with trisomy 16 (n = 4), trisomy 22 (n = 2), and a normal karyotype (n = 4).

One of the analyzed hypermethylated genes, *GATA3-AS1*, enhances the expression of the key transcription factor GATA3, which controls trophoblast differentiation^13^. Therefore, we compared the expression of the GATA3 protein in chorionic villi of miscarriages with trisomy 16, trisomy 22, and normal karyotypes. The level of GATA3 protein expression was reduced in miscarriages with trisomy 16 compared to miscarriages with trisomy 22, and miscarriages with a normal karyotype had an intermediate level of GATA3 expression (Fig. 5).

## Discussion

Only one previous study examined the methylation profile in fetuses with trisomy 16 in the first and third trimesters of pregnancy, in which disturbances of placental DNA methylation in the third trimester and the association of aberrant methylation of certain genes with early-onset preeclampsia were studied in detail^9^. However, changes in genome methylation in the placentas of miscarriages in the first trimester were not fully analyzed.

We reviewed our earlier data on the analysis of genome-wide methylation of chorionic villi of first-trimester miscarriages with trisomy 16 and induced abortions using the Infinium HumanMethylation27 BeadChip (Illumina)^14^. Our results confirmed earlier findings that trisomy 16 was associated with an increase in the methylation index of several genes in the chorionic villi, and this increased methylation may play a role in embryo death in the first trimester of pregnancy.

The presence of significant correlations between increased methylation of individual gene promoters and genome hypermethylation, and the fact that the LINE-1 retrotransposon methylation index is increased in the chorionic villi of miscarriages with trisomy 16 compared to induced abortions^15^, indicate that the hypermethylation of DNA in trisomy 16 may be specific to the whole genome. This process is likely triggered by specific gene(s) that are located on chromosome 16. These genes may be involved in the regulation of DNA methylation. According to the Gene Ontology database, several genes involved in DNA and histone methylation are located on chromosome 16. These include four histone methyltransferases (SETD6, SETD1A, PRDM7, and PRMT7), methyltransferase coactivators (CREBBP and PAGR1), and the key chromatin insulator CTCF. Histone and DNA methylation are often linked in repetitive centromeric and subcentromeric sequences^16^, but it is difficult to explain how an increase in the number of genes of histone methyltransferases and their cofactors on chromosome 16 induce an increase in the level of DNA methylation in gene promoters.

The present study found an increase in the DMG methylation index in miscarriages with pure and mosaic trisomy, regardless of the proportion of aneuploid clone cells, which indicates the simultaneous generation of an increased methylation index of the studied genes in the zygote or at the cleavage stage due to a disturbance of the normal mechanisms of hypomethylation^17^. The correlation of the methylation index of some gene regions with gestational age suggests that the increase in the methylation index of the studied genes is not a one-time event but develops gradually and increases with the age of the embryo. The level of DNA methylation may increase on a background of trisomy in gametes or during early development then further increases during the course of pregnancy. This assumption is supported by the increase in the level of genome methylation over the course of normal pregnancy^12^. An earlier study also found increased levels of gene promoter methylation in the placentas of fetuses with trisomy 16, and this increase was more significant than fetuses with a normal karyotype. Eighty-five hypermethylated sites were identified in the placentas of first-trimester trisomy 16 embryos, and approximately 1,195 CpG sites were identified in the third-trimester placentas compared to placentas with a normal karyotype^9^.

Among the hypermethylated genes in chorionic villi of miscarriages with trisomy 16, the *GATA3-AS1* gene is particularly interesting. This lncRNA gene is adjacent to GATA3 and is a positive regulator of GATA3 transcription^13^. GATA3 is a transcription factor that is selectively expressed in the trophectoderm at the blastocyst stage of embryo development. GATA3 knockdown leads to a significant decrease in migration and a decrease in HTR8/SVneo cell invasion^18^. GATA3 is essential for the development of trophectoderm clones and maintaining the self-renewal of trophoblastic stem cells^19^. Together with GATA2, GATA3 is involved in the regulation of the α subunit of chorionic gonadotropin, syncytin, HLA-G, and several other genes in human trophoblast cells^19^. DNA methylation regulates the *GATA3* gene during human embryonic stem cell differentiation^20^. However, its methylation status during trophoblast differentiation is not clear. Due to the limited available material of miscarriages in our study, only the DNA methylation profile was studied without analysis of the transcription profile of genes. Therefore, we confirmed the association of GATA3-AS1 hypermethylation and reduced GATA3 protein levels in chorionic villi sections in miscarriages with trisomy 16 compared to controls.

The genes of various secreted factors, their receptors, and signal peptides were predominantly hypermethylated in the chorionic villi of miscarriages with trisomy 16 in our study. These genes included *TRPV6*, *SLC13A4*, and *CALCB*. The *TRPV6* gene encodes a member of a family of multipass membrane proteins that function as calcium channels. *TRPV6* is expressed in the human placenta, primarily in trophoblasts^21^, and its expression in the placenta and fetal bone tissue persists throughout mouse pregnancy^22^. The *CALCB* gene encodes calcitonin-related beta-peptide (CGRP), which is a neurotransmitter. Its receptors were identified on human trophoblast cells. CGRP is involved in the morphological and functional differentiation of trophoblast cells via CGRP-induced accumulation of intracellular cAMP^23^. SLC13A4 is one of the most common sulfate transporters, and it is expressed primarily in the placenta in syncytiotrophoblast and cytotrophoblast cells^24^. Knockout of this gene in mouse embryos leads to severe fetal abnormalities and prenatal death^25^. Hypermethylation of these genes may lead to inhibition of their expression and disturbance of the normal development and differentiation of the trophoblast, death of embryos with pure trisomy 16 in the early stages of development, or malformations of fetuses with mosaic forms of trisomy 16 that survive until the third trimester.

Mosaic trisomy 16 is a consequence of trisomy rescue, a phenomenon with unclear causes. Aneuploidy itself causes chromosomal instability because of defects in cell cycle progression^26^ or cell proliferation^27^. The level of anaphase lagging is significantly higher in cells with trisomy 7 or 13 than diploid cells due to errors in cytokinesis. This phenomenon is associated with an increase in the concentration of the spartin protein, which is localized in centrosomes and microtubules during mitosis. The *SPG20* gene encoding this protein is located on chromosome 13^28^.

For trisomy 16, we found hypermethylation of two genes that are involved in mitosis, *ANKRD53* and *CDKN1A* (Supplementary Table S1). ANKRD53 protein localizes to the microtubules of the mitotic spindle around the centrosome in prophase and prometaphase and at the spindle poles in metaphase and anaphase, and it regulates the polymerization of microtubules and cytokinesis^29^. Defects in the methylation of the *ANKRD53* gene may lead to an increase in the frequency of anaphase lagging of various chromosomes and not only supernumerary chromosomes. The second hypermethylated gene, *CDKN1A*, encodes an inhibitor of the cyclin-dependent kinase p21. Increased expression of p21 serves as a cellular defense mechanism for suppressing further chromosomal instability and tumor development, and reduced expression following *CDKN1A* knockout leads to chromosomal instability^30^. Therefore, hypermethylation of this gene may also initiate the process of trisomy rescue.

The conclusions of our study have some limitations related to the tools used, the study design and the stages of development studied. The methylation index of CpG sites was analyzed only in chorionic villi, and the results obtained cannot be extrapolated to embryo tissues. Because only miscarriages at 6–10 weeks of development were included in our study, we could not distinguish between epigenetic disorders that originate in earlier stages of embryonic development, such as demethylation errors at the cleavage stage or when establishing a tissue-specific methylation pattern. The platform used for differential methylation analysis also imposes some limitations. The Illumina Infinium HumanMethylation27 BeadChip platform has a nonuniform genome coverage density, and the identified differentially methylated CpG sites are predominantly located in the promoter regions of genes. Therefore, the identification of the methylation profile in intergenic regions and gene bodies requires further studies using bisulfite whole genome sequencing. The identification of differentially methylated sites in genes does not always reflect changes in their expression and requires additional verification using functional approaches. The present study only analyzed changes in the expression of the GATA3 gene. Functional studies of the role of other identified differentially methylated genes in embryonic development will help elucidate the mechanisms of normal interactions between the embryo and the mother in the first trimester of pregnancy.

The present study confirmed that trisomy 16 was associated with large-scale methylation defects in chorionic villi and identified genes with aberrant methylation that may disrupt normal trophoblast differentiation and invasion and the normal mother-fetus interaction. We also identified genes that may initiate the process of mitotic instability and correction of trisomy 16.

## Material and methods

### Materials and karyotyping

We used samples of chorionic villi from 14 first-trimester miscarriages with trisomy 16 and 6 induced abortions, for which a genome-wide analysis of methylation using the Infinium HumanMethylation27 BeadChip (Illumina, USA) was previously performed (Supplementary Table S2)^14^. The methylation index of individual genes was analyzed in chorionic villi of 29 first-trimester miscarriages with trisomy 16 (Supplementary Table S2) using targeted bisulfite massive parallel sequencing. The DNA methylation index was also analyzed in 22 miscarriages with other aneuploidies, 7 miscarriages with normal karyotypes, and 8 induced abortions (Table 3).

**Table 3:** Characteristics of miscarriages with a normal karyotype, miscarriages with aneuploidy and induced abortions.

Group	Aneuploidy	Microarray analysis		Targeted bisulfite massive parallel sequencing	
		Sample size	Gestational age, mean ± SD, (min–max), w	Sample size	Gestational age, mean ± SD, (min–max), w
Miscarriages with trisomy 16	Trisomy 16	12	8.4 ± 2.0 (6–12)	29	9.4 ± 1.8 (6–12)
Miscarriages with other aneuploidy	See Table S2	–	–	22	8.9 ± 1.4 (6.5–11.5)
Miscarriages with a normal karyotype	–	–	–	7	10.1 ± 3.6 (6.5–16.5)
Induced abortions	–	6	8.8 ± 1.9 (7–12)	8	9.4 ± 2.7 (7–12)

Chorionic villi were separated from other extraembryonic and maternal tissues via morphology under the control of an inverted microscope. A suspension of cells was prepared from the chorionic villi samples, part of which was used to confirm the karyotype using FISH analysis, and DNA was extracted from the other part to determine the level of DNA methylation. Therefore, FISH analysis and DNA methylation analysis were performed from a common mixture of cells and not from different sections of the chorionic villi.

All of the women signed informed consent forms. The local Research Ethics Committee of the Research Institute of Medical Genetics granted their approval (22.04.2010/No 2). Before the start of the study, the samples were stored at − 80 °C without thawing. For all miscarriages, conventional cytogenetic analysis was performed on trophoblast cells or cultured extraembryonic fibroblasts, as described previously^31^. When the conventional cytogenetic analysis was noninformative, karyotypes were assessed using chromosomal comparative genome hybridization (cCGH, n = 6) or array comparative genome hybridization (aCGH, n = 4) (Fig. 1) as described elsewhere^15^.

### FISH

The karyotyping results in 29 miscarriages with trisomy 16 were validated using interphase FISH. The degree of mosaicism of trisomy 16 was assessed simultaneously with lower and upper thresholds of 10% and 90%, respectively. Two-color subtelomeric (16p and 16q) DNA probes were used for analysis (Fig. 1). DNA probes were kindly provided by Prof. Mariano Rocchi at the University of Bari, Italy. FISH was performed as previously described^32^. Thirteen miscarriages had mosaicism of trisomy 16 (aneuploid clone—from 10 to 89%), and 16 miscarriages had pure trisomy 16 (aneuploid clone ≥ 90%).

### Genome-wide DNA methylation analysis

Chorionic villous trophoblast cells were enriched via maceration of chorionic villi in 70% acetic acid using a modified protocol^33^. The isolated cells were washed in phosphate buffer. Genomic DNA was isolated from chorionic villi using the phenol–chloroform method. DNA methylation profiles were determined using the Infinium HumanMethylation27 BeadChip (Illumina, USA), which includes 27,578 CpG sites in 14,475 genes, according to the manufacturer's protocol. The data were analyzed using the software package GenomeStudio Methylation Module (Illumina), which translates the fluorescence intensity into a quantitative value of β (methylation index) that corresponds to the ratio of the fluorescence signals of methylated alleles to the sum of the signals of methylated and unmethylated alleles of the studied locus. The absolute difference in the means of the β values between compared groups was calculated for each CpG within a region and referred to as the delta beta (Δβ) value.

Analysis of DNA methylation microarray data was performed using the Bioconductor lumi^34^ and limma^35^ packages in the R statistical environment. Briefly, color bias correction and quantile normalization were used for data preprocessing. Differentially methylated CpG sites in chorionic villi between miscarriages with trisomy 16 and induced abortions were assessed using an empirical Bayes moderated t-test^36^. The Benjamini–Hochberg test was used to adjust the p values for the FDR. Enrichment analysis of the list of genes with differentially methylated CpG sites was performed using DAVID software^37^. Functional relationships between DMGs and genes involved in placental development were analyzed using the STRING tool^38^.

### Targeted bisulfite massive parallel sequencing

The DNA methylation index in the promoter regions of 5 genes (*ANKRD53, TRPV6, GATA3-AS1, SCL13A4,* and *CALCB*) was measured using bisulfite amplicon massive parallel sequencing. Sodium bisulfite conversion of genomic DNA was performed using an EZ DNA Methylation-Direct Kit (Zymo Research, USA). After bisulfite conversion of DNA, amplification of the products was performed using PCR with self-designed primers (Supplementary Table S3). The primers were designed using MethPrimer^39^, and their specificity was assessed using the BiSearch tool^40^. PCR was performed with BioMaster HS-Taq PCR-Color (2 ×) PCR mix (BioLabMix, Russia). The following PCR conditions were used: primary denaturation, 15 min at 95 °C; 45 cycles of 20 s at 95 °C, 20 s at 50 °C, and 20 s at 72 °C; and terminal elongation, 5 min at 72 °C.

Amplified DNA was isolated using Sephadex G50 (Sigma, USA) and pooled for library generation. Adapters and indices were attached using the Nextera XT kit (Illumina) according to the manufacturer’s protocol. Sequencing was performed on a MiSeq sequencer using the MiSeq Reagent Micro Kit v2 kit (Illumina, San Diego, CA, USA) according to the manufacturer’s protocol.

For analysis of the targeted bisulfite sequencing results, the obtained reads were filtered in the Trimmomatic program with a Phred threshold of 30. The filtered reads were aligned to in silico bisulfite-converted DNA in BWA-MEM. We counted the number of aligned nucleotides at each position and calculated the methylation index, the proportion of methylated cytosines, with SAMtools and R using the formula Methyl = C/(C + T). The distribution of data was analyzed using the Kolmogorov–Smirnov test. The Mann–Whitney U test was used to compare groups. The Spearman test was used for correlation analyses.

### Immunohistochemistry

Seven-micrometer-thick sections of frozen samples of chorionic villi of miscarriages with trisomy 16 (n = 4), trisomy 22 (n = 2), and normal karyotype (n = 4) stored in liquid nitrogen were mounted on poly-l-lysine slides (Thermo Fisher Scientific, Waltham, MA, USA). Briefly, the sections were fixed in paraformaldehyde (Sigma, St. Louis, MO, USA) for 5 min and washed twice in phosphate-buffered saline (PBS) for 5 min. The sections were incubated for 5 min with a peroxidase blocking reagent (Agilent, Santa Clara, CA, USA) and washed twice in PBS for 5 min. The sections were incubated for 15 min with a primary anti-GATA3 antibody (EP368, 1:100, Merck KGaA, Darmstadt, Germany) and washed twice in PBS for 5 min. The sections were incubated for 15 min with EnVision Flex/HRP (Agilent, Santa Clara, CA, USA), washed twice in PBS for 5 min, and incubated for 5 min with a diaminobenzidine (DAB) solution (Agilent, Santa Clara, CA, USA). Nuclei were contrasted using hematoxylin. The sections were mounted according to a standard operating procedure. As a control for nonspecific binding, sections were incubated with an appropriate primary antibody or with only the secondary antibody. Slides were scanned using an Aperio AT2 (Leica, Wetzlar, Germany). Image analysis was performed using the ImageJ program using the IHC Profiler plugin^41,42^.

### Ethics declarations

This study was performed in accordance with the principles of the Declaration of Helsinki. The local Research Ethics Committee of the Research Institute of Medical Genetics, Tomsk NRMC approved this study (22.04.2010/No 2).

## Supplementary Information

Supplementary Information.

## Data Availability

The datasets used and/or analyzed during the current study are available from the corresponding author on request.
