# Supplementary material for: Identification of differentially methylated genes in first-trimester placentas with trisomy 16
Source: Sci Rep. 2022 Jan 21;12:1166. doi: 10.1038/s41598-021-04107-9 (PMC8782849; doi:10.1038/s41598-021-04107-9)
Supplement: Supplementary file 1 — Supplementary Information. [file 41598_2021_4107_MOESM1_ESM.pdf]

Table S1. Differentially methylated sites (FDR cutoff of $p<0.05$ and $\Delta\beta>0.15$) in chorionic villi of miscarriages with trisomy 16

TargetID	Beta IA	Beta tri16	DeltaB	Chr	Gene name	Product	CpG island	Location
cg23219720	0.48	0.3	0.18	7	*ZNF804B*	zinc finger protein 804B	FALSE	intron 1
cg27319898	0.5	0.22	0.28	7	*ZNF804B*	zinc finger protein 804B	FALSE	promoter
cg18006568	0.28	0.55	-0.28	2	*ANKRD53*	ANKRD53 ankyrin repeat domain 53	FALSE	intron 1
cg02786019	0.18	0.45	-0.27	7	*TRPV6*	transient receptor potential cation channel; subfamily V; member 6	FALSE	promoter
cg20831708	0.27	0.53	-0.26	10	*SEC31L2*	S. cerevisiae SEC31-like 2 isoform a	FALSE	promoter
cg21109025	0.4	0.64	-0.23	17	*CCL2*	small inducible cytokine A2 precursor	FALSE	exon 1
cg04765277	0.17	0.4	-0.23	10	*GATA3-AS1*	GATA3 antisense RNA 1	FALSE	promoter
cg02005755	0.23	0.45	-0.22	7	*SLC13A4*	solute carrier family 13 (sodium/sulfate symporters); member 4	FALSE	5'-UTR
cg21627181	0.44	0.66	-0.22	6	*SLC17A4*	solute carrier family 17 (sodium phosphate); member 4	FALSE	exon 1
cg18236297	0.43	0.65	-0.22	13	*CYSLTR2*	cysteinyl leukotriene receptor 2	TRUE	exon 1
cg08109646	0.42	0.63	-0.22	1	*ZNF683*	zinc finger protein 683	FALSE	promoter
cg06938878	0.44	0.66	-0.21	11	*CALCB*	calcitonin-related polypeptide; beta	TRUE	exon 1
cg05461276	0.38	0.59	-0.21	11	*PDZD3*	natrium-phosphate cotransporter IIa C-terminal-associated protein 2	FALSE	5'-UTR
cg26647600	0.47	0.68	-0.21	3	*CCR8*	chemokine (C-C motif) receptor 8	FALSE	promoter
cg05293216	0.22	0.43	-0.21	9	*FANCG*	Fanconi anemia; complementation group G	FALSE	down-stream
cg17778867	0.47	0.67	-0.2	21	*KRTAP10-8*	keratin associated protein 10-8	FALSE	intron 1
cg15016628	0.18	0.39	-0.2	X	*BRS3*	bombesin-like receptor 3	FALSE	promoter
cg07269146	0.41	0.61	-0.2	9	*CCL21*	small inducible cytokine A21 precursor	FALSE	promoter
cg02192965	0.38	0.58	-0.2	2	*SLC3A1*	solute carrier family 3; member 1	FALSE	promoter
cg21283680	0.48	0.68	-0.2	3	*SH3BP5*	SH3-domain binding protein 5 (BTK-associated) isoform a	FALSE	exon 1
cg18959422	0.29	0.49	-0.2	1	*MYBPH*	myosin binding protein H	FALSE	exon 1
cg00152644	0.54	0.74	-0.2	1	*SPRR2E*	small proline-rich protein 2E	FALSE	promoter
cg20312687	0.31	0.5	-0.2	20	*DEFB118*	defensin; beta 118	FALSE	exon 1
cg18818531	0.14	0.33	-0.19	11	*FOSL1*	FOS-like antigen 1	TRUE	5'-UTR
cg17502536	0.56	0.75	-0.19	1	*PADI1*	peptidylarginine deiminase type I	TRUE	5'-UTR
cg26661623	0.5	0.69	-0.19	17	*ASGR2*	asialoglycoprotein receptor 2 isoform a	FALSE	promoter
cg10895543	0.19	0.38	-0.19	9	*CDKN2A*	cyclin-dependent kinase inhibitor 2A isoform 3	TRUE	5'-UTR
cg21788470	0.29	0.47	-0.19	12	*PTHLH*	parathyroid hormone-like hormone isoform 2 preproprotein	FALSE	promoter
cg14183455	0.44	0.63	-0.19	1	*MATN1*	matrilin 1; cartilage matrix protein	FALSE	promoter
cg16192029	0.59	0.77	-0.18	7	*ANKRD7*	testis-specific ankyrin motif containing protein	FALSE	promoter
cg20672044	0.56	0.74	-0.18	16	*HPR*	haptoglobin-related protein	FALSE	promoter
cg24325790	0.5	0.68	-0.18	17	*CCL23*	small inducible cytokine A23 isoform CKbeta8-1 precursor	FALSE	promoter
cg04616566	0.44	0.62	-0.18	15	*THSD4*	hypothetical protein LOC79875	FALSE	promoter
cg18885346	0.2	0.38	-0.18	6	*PKHD1*	polyductin isoform 1	TRUE	exon 1
cg12455187	0.45	0.63	-0.18	17	*CCL5*	small inducible cytokine A5 precursor	TRUE	5'-UTR
cg09699159	0.44	0.62	-0.18	11	*PRDM11*	PR domain containing 11	TRUE	promoter
cg04979933	0.28	0.46	-0.18	1	*INSL5*	insulin-like 5 precursor	FALSE	exon 1

cg15792367	0.31	0.48	-0.18	19	*KLK11*	kallikrein 11 isoform 1 preproprotein	FALSE	exon 1
cg19690101	0.52	0.7	-0.18	2	*DHX57*	DEAH (Asp-Glu-Ala-Asp/His) box polypeptide 57 isoform 3	TRUE	promoter
cg24993443	0.29	0.47	-0.18	15	*SNRPN*	small nuclear ribonucleoprotein polypeptide N	FALSE	exon 1
cg15737168	0.48	0.65	-0.18	11	*TECTA*	tectorin alpha precursor	TRUE	exon 1
cg18110483	0.15	0.32	-0.17	5	*THBS4*	thrombospondin 4 precursor	FALSE	promoter
cg15916061	0.26	0.44	-0.17	6	*SLC17A4*	solute carrier family 17 (sodium phosphate); member 4	FALSE	intron 1
cg07142319	0.47	0.64	-0.17	1	*PLA2G2D*	phospholipase A2; group IID	TRUE	promoter
cg13180098	0.54	0.71	-0.17	3	*RHO*	rhodopsin	FALSE	promoter
cg22568540	0.58	0.76	-0.17	19	*A1BG*	alpha 1B-glycoprotein	TRUE	intron 2
cg19949550	0.54	0.72	-0.17	14	*ASB2*	ankyrin repeat and SOCS box-containing protein 2	FALSE	exon 1
cg27378216	0.51	0.69	-0.17	18	*SETBP1*	SET binding protein 1	FALSE	promoter
cg21644628	0.58	0.75	-0.17	16	*HEATR3*	HEAT repeat containing 3	TRUE	intron 1
cg05501357	0.34	0.51	-0.17	11	*HIPK3*	homeodomain interacting protein kinase 3	TRUE	exon 1
cg11812202	0.38	0.55	-0.17	10	*PNLIP*	pancreatic lipase precursor	FALSE	intron 1
cg22815534	0.22	0.38	-0.17	22	*CECR5*	cat eye syndrome chromosome region; candidate 5 isoform 1	TRUE	intron 2
cg04797496	0.47	0.64	-0.17	5	*PCDH12*	protocadherin 12 precursor	TRUE	intron 2
cg20610181	0.49	0.66	-0.17	9	*CA9*	carbonic anhydrase IX precursor	TRUE	intron 4
cg17854440	0.19	0.36	-0.17	4	*ENPEP*	glutamyl aminopeptidase (aminopeptidase A)	FALSE	promoter
cg14345676	0.53	0.7	-0.17	5	*HRH2*	histamine receptor H2	TRUE	promoter
cg02921257	0.54	0.71	-0.17	3	*CMYA1*	cardiomyopathy associated 1	FALSE	up-stream
cg02046017	0.52	0.69	-0.17	11	*SHANK2-AS3*	SHANK2 antisense RNA 3	TRUE	promoter
cg09407859	0.6	0.77	-0.17	16	*CES3*	carboxylesterase 3	FALSE	promoter
cg03271907	0.64	0.8	-0.17	10	*MGMT*	O-6-methylguanine-DNA methyltransferase	FALSE	intron 1
cg00323915	0.52	0.68	-0.16	7	*GIMAP4*	GTPase; IMAP family member 4	FALSE	exon 2
cg17463527	0.36	0.52	-0.16	20	*SGK2*	serum/glucocorticoid regulated kinase 2 isoform beta	FALSE	promoter
cg03882305	0.2	0.37	-0.16	7	*TRIM50C*	hypothetical protein LOC378108	FALSE	exon 2
cg04739149	0.34	0.5	-0.16	3	*DOC1*	downregulated in ovarian cancer 1 isoform 1	FALSE	intron 2
cg10333959	0.61	0.78	-0.16	10	*MGMT*	O-6-methylguanine-DNA methyltransferase	TRUE	intron 1
cg26705561	0.33	0.49	-0.16	10	*SEC31L2*	S. cerevisiae SEC31-like 2 isoform a	TRUE	intron 1
cg07131544	0.34	0.5	-0.16	6	*NCR2*	natural cytotoxicity triggering receptor 2	FALSE	promoter
cg00014837	0.59	0.75	-0.16	12	*ACRBP*	proacrosin binding protein sp32 precursor	FALSE	promoter
cg22802439	0.34	0.5	-0.16	19	*UPK1A*	uroplakin 1A	TRUE	intron 23
cg09450238	0.21	0.37	-0.16	14	*BTBD6*	BTB domain protein BDPL	FALSE	exon 1
cg00412805	0.54	0.7	-0.16	3	*KBTBD5*	kelch repeat and BTB (POZ) domain containing 5	FALSE	exon 1
cg06956273	0.46	0.62	-0.16	1	*GNAT2*	guanine nucleotide binding protein; alpha transducing activity polypeptide 2	FALSE	5'-UTR
cg01703884	0.58	0.73	-0.16	12	*FLJ37587*	myosin 1H	TRUE	exon 4
cg15727249	0.54	0.7	-0.16	11	*APOA4*	apolipoprotein A-IV precursor	TRUE	promoter
cg27655855	0.42	0.58	-0.16	20	*CST9L*	cystatin 9-like precursor	FALSE	exon 2
cg27600794	0.55	0.71	-0.16	10	*PNLIPRP1*	pancreatic lipase-related protein 1	TRUE	intron 2

cg03459809	0.44	0.59	-0.16	1	*EPHX1*	epoxide hydrolase 1; microsomal (xenobiotic)	FALSE	promoter
cg14722162	0.42	0.58	-0.16	5	*C5orf20*	dendritic cell nuclear protein 1	FALSE	promoter
cg10919204	0.21	0.37	-0.16	5	*CDH6*	cadherin 6; type 2 preproprotein	TRUE	intron 1
cg04744379	0.39	0.55	-0.16	19	*KLK15*	kallikrein 15 isoform 1 preproprotein	FALSE	promoter
cg05810550	0.48	0.64	-0.16	8	*DEFB106A*	defensin; beta 106A	FALSE	promoter
cg07597976	0.37	0.53	-0.16	16	*CD19*	CD19 antigen	FALSE	promoter
cg16158220	0.51	0.66	-0.16	2	*REG3A*	Regenerating Islet-Derived Protein 3-Alpha	FALSE	promoter
cg13944141	0.33	0.49	-0.16	7	*PRSS2*	protease; serine; 2 preproprotein	FALSE	promoter
cg10979891	0.24	0.4	-0.15	14	*SMOC1*	secreted modular calcium-binding protein 1	FALSE	exon 2
cg04595372	0.24	0.39	-0.15	6	*CRISP2*	cysteine-rich secretory protein 2	FALSE	promoter
cg25995212	0.37	0.53	-0.15	2	*SCN7A*	sodium channel; voltage-gated; type VII; alpha	FALSE	intron 2
cg01970325	0.27	0.42	-0.15	9	*NELF*	nasal embryonic LHRH factor	FALSE	intron 1
cg11286122	0.32	0.47	-0.15	11	*PHLDB1*	pleckstrin homology-like domain; family B; member 1	FALSE	promoter
cg24477636	0.58	0.74	-0.15	19	*OR10H1*	olfactory receptor; family 10; subfamily H; member 1	FALSE	promoter
cg19526600	0.46	0.61	-0.15	1	*DIO1*	thyroxine deiodinase type 1 isoform a	FALSE	5'-UTR
cg04523589	0.6	0.75	-0.15	3	*CAMP*	cathelicidin antimicrobial peptide	FALSE	intron 1
cg13693652	0.57	0.73	-0.15	1	*CLCA2*	calcium activated chloride channel 2	FALSE	promoter
cg08914623	0.09	0.25	-0.15	11	*ALX4*	aristaless-like homeobox 4	FALSE	5'-UTR
cg01714932	0.39	0.54	-0.15	12	*PZP*	pregnancy-zone protein	FALSE	5'-UTR
cg00061629	0.37	0.52	-0.15	11	*ALX4*	aristaless-like homeobox 4	FALSE	5'-UTR

Table S2. Extended description of miscarriages with a normal karyotype, miscarriages with aneuploidy and induced abortions

Sample ID	Karyotype	Maternal age, y	Paternal age, y	Gestational age, w	Chr	Sex	Group	Analysis
tri16-1	47,XX,+16/46,XX	24	24	7.9	16	f	Tri16	BeadChip
tri16-2	47,XY,+16/46,XY	34	33	6	16	m	Tri16	BeadChip
tri16-3	47,XX,+16/46,XX	20	-	10	16	f	Tri16	BeadChip
tri16-4	47,XX,+16/46,XX	33	38	-	16	f	Tri16	BeadChip
tri16-5	47,XY,+16/46,XY	33	34	7.3	16	m	Tri16	BeadChip
tri16-6	47,XY,+16/46,XY	28	32	8.9	16	m	Tri16	BeadChip
tri16-7	47,XY,+16/46,XY	40	42	12.3	16	m	Tri16	BeadChip
tri16-8	47,XX,+16/46,XX	24	24	7.9	16	f	Tri16	BeadChip
tri16-9	47,XX,+16/46,XX	27	30	6	16	f	Tri16	BeadChip
tri16-10	47,XY,+16/46,XY	40	36	9.3	16	m	Tri16	BeadChip
tri16-11	47,XY,+16/46,XY	36	39	6.6	16	m	Tri16	BeadChip
tri16-12	47,XY,+16/46,XY	34	30	10.5	16	m	Tri16	BeadChip
tri16-13	47,XY+16	34	33	6	16	m	Tri16	Targeted NGS
tri16-14	47,XY+16	21	-	6	16	m	Tri16	Targeted NGS
tri16-15	47,XX+16	20	-	10	16	f	Tri16	Targeted NGS
tri16-16	47,XY+16	33	34	12	16	m	Tri16	Targeted NGS
tri16-17	47,XY,+16/46,XY	24	24	8	16	f	Tri16	Targeted NGS
tri16-18	47,XY+16	34	30	10.5	16	m	Tri16	Targeted NGS
tri16-19	47,XY+16	29	29	8.6	16	m	Tri16	Targeted NGS
tri16-20	47,XX+16	20	-	10	16	f	Tri16	Targeted NGS
tri16-21	47,XX+16	28	31	8.5	16	f	Tri16	Targeted NGS
tri16-22	47,XX+16	32	39	8	16	f	Tri16	Targeted NGS
tri16-23	47,XY+16	28	31	11	16	m	Tri16	Targeted NGS
tri16-24	47,XY+16	31	25	12	16	m	Tri16	Targeted NGS
tri16-25	47,XY+16	29	30	12	16	m	Tri16	Targeted NGS
tri16-26	47,XY+16	27	29	9.0	16	m	Tri16	Targeted NGS
tri16-27	47,XX+16	37	41	9.0	16	m	Tri16	Targeted NGS
tri16-28	47,XY+16	31	33	10.0	16	m	Tri16	Targeted NGS
tri16-29	47,XY+16/46,XY	33	36	9.0	16	m	Tri16	Targeted NGS
tri16-30	47,XX+16	25	34	9.5	16	f	Tri16	Targeted NGS
tri16-31	47,XY+16	22	27	11.2	16	m	Tri16	Targeted NGS
tri16-32	47,XY+16/46,XY	27	28	9.0	16	m	Tri16	Targeted NGS
tri16-33	47,XY+16	35	29	9.5	16	m	Tri16	Targeted NGS
tri16-34	47,XX+16	26	31	9	16	f	Tri16	Targeted NGS
tri16-35	47,XX+16	-	-	8.5	16	m	Tri16	Targeted NGS
tri16-36	47,XY+16	39	29	11.0	16	m	Tri16	Targeted NGS
tri16-37	47,XX+16	-	-	11.0	16	m	Tri16	Targeted NGS
tri16-38	47,XX+16	33	38	7	16	f	Tri16	Targeted NGS
tri16-39	47,XY+16	28	28	8.2	16	m	Tri16	Targeted NGS
tri16-40	47,XX+16	37	42	11	16	f	Tri16	Targeted NGS
tri16-41	47,XY+16/46,XY	24	32	9	16	m	Tri16	Targeted NGS
IA-1	46,XY	35	-	8	-	m	IA	BeadChip

IA-2	46,XY	24	-	7	-	m	IA	BeadChip
IA-3	46,XX	28	-	10	-	f	IA	BeadChip
IA-4	46,XX	27	-	7	-	f	IA	BeadChip
IA-5	46,XX	33	-	9	-	f	IA	BeadChip
IA-6	46,XY	36	-	12	-	m	IA	BeadChip
IA-7	46,XY	24	-	11	-	m	IA	Targeted NGS
IA-8	46,XY	29	-	11.5	-	m	IA	Targeted NGS
IA-9	46,XX	44	-	12	-	f	IA	Targeted NGS
IA-10	46,XX	24	-	7	-	f	IA	Targeted NGS
IA-11	46,XX	25	-	8	-	f	IA	Targeted NGS
IA-12	46,XY	30	-	9	-	m	IA	Targeted NGS
IA-13	46,XY	38	-	10	-	m	IA	Targeted NGS
IA-14	46,XY	35	-	7	-	m	IA	Targeted NGS
Misc-1	47,XY+21	35	48	11.5	21	m	Misc	Targeted NGS
Misc-2	47,XX+14	23	33	8	14	f	Misc	Targeted NGS
Misc-3	47,XX+15	39	37	11.0	15	f	Misc	Targeted NGS
Misc-4	47,XX+2	21	25	6.5	2	f	Misc	Targeted NGS
Misc-5	47,XX+15	38	31	8.5	15	f	Misc	Targeted NGS
Misc-6	47,XY+18	38	26	11.5	18	m	Misc	Targeted NGS
Misc-7	47,XX,-13/46,XX	22	-	7	13	f	Misc	Targeted NGS
Misc-8	47,XX+2	27	27	9	2	f	Misc	Targeted NGS
Misc-9	47,XX+13	20	-	9	13	f	Misc	Targeted NGS
Misc-10	47,XY+2	39	40	9.5	2	m	Misc	Targeted NGS
Misc-11	47,XX+7	27	27	8	7	f	Misc	Targeted NGS
Misc-12	45,XY-15/46,XY	23	22	10.5	X	m	Misc	Targeted NGS
Misc-13	47,XY+7/46,XY	24	22	10.5	7	m	Misc	Targeted NGS
Misc-14	47,XX+9	43	45	8	9	f	Misc	Targeted NGS
Misc-15	47,XX+20	31	32	8	20	f	Misc	Targeted NGS
Misc-16	47,XX+9	26	32	7	9	f	Misc	Targeted NGS
Misc-17	45,X/46,XX	21	23	10.5	X	f	Misc	Targeted NGS
Misc-18	47,XY+9/46,XY	32	32	7.6	9	m	Misc	Targeted NGS
Misc-19	47,XX+11	38	33	7	11	f	Misc	Targeted NGS
Misc-20	47,XX+22	32	37	8	22	f	Misc	Targeted NGS
Misc-21	47,XY+18/46,XY	29	29	10	18	m	Misc	Targeted NGS
Misc-22	47,XX+13	37	37	9	13	f	Misc	Targeted NGS
MNK-1	46,XX	27	-	16.5	-	f	MNK	Targeted NGS
MNK-2	46,XY	24	-	7.5	-	m	MNK	Targeted NGS
MNK-3	46,XY	28	28	7.8	-	m	MNK	Targeted NGS
MNK-4	46,XY	26	29	12.0	-	m	MNK	Targeted NGS
MNK-5	46,XY	35	34	13.0	-	m	MNK	Targeted NGS
MNK-6	46,XY	31	33	10.7	-	m	MNK	Targeted NGS
MNK-7	46,XY	26	25	6.5	-	m	MNK	Targeted NGS
MNK-8	46,XY	31	-	6.8	-	m	MNK	Targeted NGS

Comments: Tri16 – miscarriages with trisomy 16, IA – induced abortions, Misc – miscarriages with aneuploidy on other chromosomes, MNK – miscarriages with normal karyotype

Table S3. Primers for targeted bisulfite massive parallel sequencing for assessment of DNA methylation in the promoters of the *ANKRD53*, *TRPV6*, *GATA3-AS1*, *SCL13A4*, and *CALCB* genes

Product	Primer sequence	Product length (bp)	CpG	Genomic coordinates (hg19)
ANKRD53_Meth1	F 5'-TTGTAATAAATTATGATTTTTGTG-3'<br>R 5'-CCCAACCTATCTCTACTAACTTCTC-3'	700	40	chr2: 71206056-71206605
CALCB_Meth_1	F 5'-TTGTTTGTGAATGTAGAAGGTAGAG-3'<br>R 5'-CCACCTATTTACCAAACTACCTAAC-3'	276	15	chr11: 15094027-15094302
CALCB_Meth_3	F 5'-AGGGTTGGTTGTTAGATTGGTATTA-3'<br>R 5'-CACCTCCTTAACTCCTAAATTCCTAC-3'	308	8	chr11: 15094516-15094823
CALCB_Meth_4	F 5'-GTAGGAATTTAGGAGTTAAGGAGGTG-3'<br>R 5'-CCAATCCAAAATAACCAAAACTAAC-3'	269	12	chr11: 15094798-15095066
TRPV6_Meth_1	F 5'-GTAGAAGAGGTATTGTGGGAAAGTG-3'<br>R 5'-CCCCAACCAATTTAAAAAAAACTATA-3'	436	5	chr7: 142583430-142583865
TRPV6_Meth_2	F 5'-TTGTTGGGAGATTTTTAAGGAATT-3'<br>R 5'-CTCTTTCACCAACCCTACAAACTAA-3'	480	11	chr7: 142582950-142583429
TRPV6_Meth_3	F 5'-GAGAAAGGGTTAATTTTTTGTTTATG-3'<br>R 5'-ATACTCTATCCTTACAAACCCCAAC-3'	411	6	chr7: 142582830-142583240
SLC13A4_Meth_1	F 5'-GTAGGGAGGTTTGAAAGGTTTAATT-3'<br>R 5'-TAAAAACCCACCTTTAACTTTCATC-3'	427	11	chr7: 135412984-135413410
SLC13A4_Meth_2	F 5'-TTTTTTATTGGTGGATGAAAGTTAAA-3'<br>R 5'-AAATATCAACAAAAACCCCCTTAAT-3'	459	7	chr7: 135412563-135413021
GATA3-AS1_Meth1	F 5'-TGTTTGAAGAGAGTTAGTTTTGTTG-3'<br>R 5'-ACCCCTAAATTAAATTACCCTAACC-3'	371	30	chr10: 8091317-8091687
GATA3-AS1_Meth2	F 5'-GAATGGGAAGGGATTTTTTTT-3'<br>R 5'-AAACTCAACCCTAAAACTACAAACC-3'	548	57	chr10: 8092469-8093016
GATA3-AS1_Meth4	F 5'-AGGGAGAATTAGGATTATTTTTTTT-3'<br>R 5'-AATATTTAACATTTAATTCCCAACC-3'	449	37	chr10: 8093670-8094118
GATA3-AS1_Meth5	F 5'-AAATTATTTAGATTAAGAAGGGTTTT-3'<br>R 5'-TCTCTCCTAAACCAACCAAACTAC-3'	586	32	chr10: 8094186-8094771
GATA3-AS1_Meth8	F 5'-TTGAATATTGTTGTTGTTTTTTTAT-3'<br>R 5'-CTTTAAACCACTACATCCCCTAAC-3'	353	15	chr10: 8095096-8095448

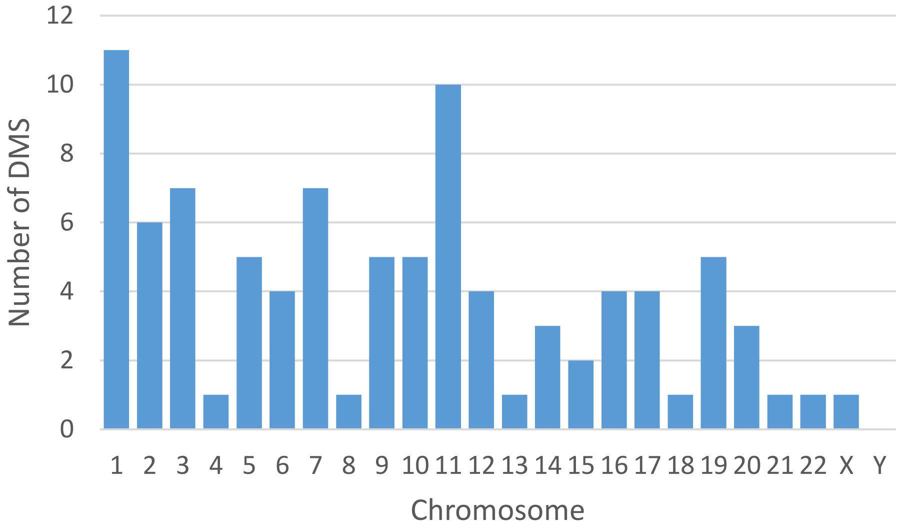

Figure S1. Distribution of differentially methylated sites by chromosomes (FDR cutoff of $p<0.05$ and $\Delta\beta>0.15$) in chorionic villi of miscarriages with trisomy 16

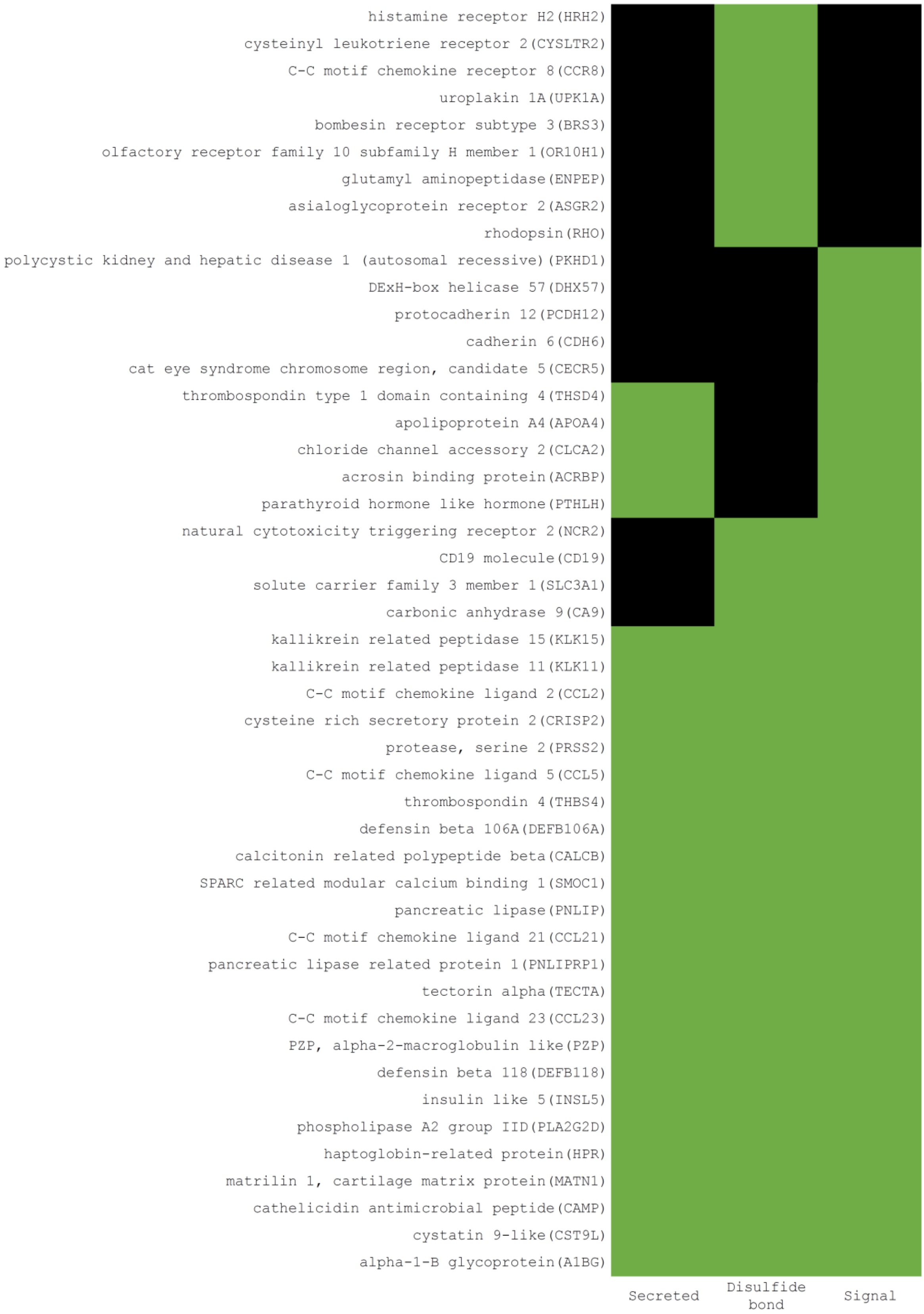

Figure S2. The significantly enriched cluster of genes encoding secreted proteins, signaling proteins, and proteins with disulfide bonds based on the results of the DAVID analysis via the UniProt database (enrichment score: 8.76). The green color indicates the inclusion of a certain gene in one of the functional groups.
